# Intra-colic textilome

**DOI:** 10.11604/pamj.2017.28.278.13616

**Published:** 2017-11-29

**Authors:** Robleh Hassan, Jesus Cabrera

**Affiliations:** 1 General Surgery Department, Balbala Hospital, Djibouti

**Keywords:** Textilome, occlusive syndrome, Gauze, Djibouti

## Image in medicine

The textilome or still called gossybipoma is a very rare but well known and not insignificant post-operative grave lesion in the abdominal and gynecological surgery. It is a foreign body compound of gauze or surgical drape omitted in the operation site. The difficulties of diagnosis delay generally the discovery of abdominal textilome. The clinicals symptoms are: chronic disorders of the transit in sub-occlusive or occlusive syndromes. Erect Abdominal X-ray has little contributory in the diagnosis, but the ultrasound remains more reliable. Abdominal CT Scan allows a precise topographic diagnosis; certain teams propose explorations by MRI. We report a case of intra-colic textilome (sigmoïde), presenting acute abdomen in forme of an occlusive syndrome in patient who was operated 3 years ago for uterine fibroid.

**Figure 1:**
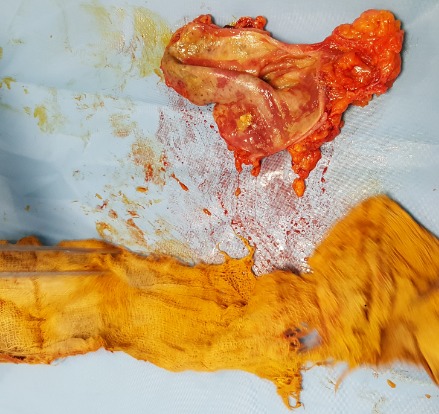
Extraction of gauze intracolic

